# A Novel Predictive Model to Estimate the Number of Mature Oocytes Required for Obtaining at Least One Euploid Blastocyst for Transfer in Couples Undergoing *in vitro* Fertilization/Intracytoplasmic Sperm Injection: The ART Calculator

**DOI:** 10.3389/fendo.2019.00099

**Published:** 2019-02-28

**Authors:** Sandro C. Esteves, José F. Carvalho, Fabiola C. Bento, Jonathan Santos

**Affiliations:** ^1^ANDROFERT, Andrology and Human Reproduction Clinic, Campinas, Brazil; ^2^Statistika Consulting, Campinas, Brazil; ^3^CliniSYS, Tecnologia e Sistemas de Saúde, Campinas, Brazil

**Keywords:** assisted reproductive technology, ART calculator, intracytoplasmic sperm injection, blastocyst, preimplantation genetic testing for aneuploidy, female age, decision support models, POSEIDON criteria

## Abstract

The POSEIDON group (**P**atient-**O**riented **S**trategies **E**ncompassing **I**ndividualize**D O**ocyte **N**umber) has introduced “the ability to retrieve the number of oocytes needed to achieve at least one euploid embryo for transfer” as an intermediate marker of successful outcome in IVF/ICSI cycles. This study aimed to develop a novel calculator to predict the POSEIDON marker. We analyzed clinical and embryonic data of infertile couples who underwent IVF/ICSI with the intention to have trophectoderm biopsy for preimplantation genetic testing for aneuploidy. We used the negative binomial distribution to model the number of euploid blastocysts and the adaptive LASSO (Least Absolute Shrinkage and Selection Operator) method for variable selection. The fitted model selected female age, sperm source used for ICSI, and the number of mature (metaphase II) oocytes as predictors (*p* < 0.0001). Female age was the most important factor for predicting the probability of a blastocyst being euploid given each mature oocyte (loglikelihood of age [adjusted for sperm source]: 30.9; *df* = 2; *p* < 0.0001). The final predictive model was developed using logistic regression analysis, and internally validated by the holdout method. The predictive ability of the model was assessed by the ROC curve, which resulted in an area under the curve of 0.716. Using the final model and mathematical equations, we calculated the individualized probability of blastocyst euploidy per mature retrieved oocyte and the minimum number of mature oocytes required to obtain ≥1 euploid blastocyst—with their 95% confidence interval [CI]—for different probabilities of success. The estimated predicted probabilities of a mature oocyte turn into a euploid blastocyst decreased progressively with female age and was negatively modulated overall by use of testicular sperm across age (*p* < 0.001). A calculator was developed to make two types of predictions automatically, one using pretreatment information to estimate the minimum number of mature oocytes to achieve ≥1 euploid blastocyst, and another based on the actual number of mature oocytes collected/accumulated to estimate the chances of having a euploid blastocyst using that oocyte cohort for IVF/ICSI. The new ART calculator may assist in clinical counseling and individualized treatment planning regarding the number of oocytes required for at least one euploid blastocyst in IVF/ICSI procedures.

## Introduction

Globally approximately 10% of the couples have difficulties to conceive, with the highest prevalence in Eastern Europe, North Africa, Middle East, and Oceania (1). Female factors, alone or combined with male factors, contribute to ~70% of infertility cases. Assisted reproductive technology (ART) has become an essential element of care for many couples suffering from infertility (2). The International Committee for Monitoring Assisted Reproductive Technologies (ICMART) reported over four million ART treatments worldwide between 2008 and 2010 (3), most of which using ICSI as the fertilization method (4). In Europe and the United States, over 2% of all infants born result from ART treatments (5), and over 8 million babies were born from ART worldwide (6).

Despite the notable developments in ART over the last decades, which improved live birth rates from 26% in the 90's to about 40% nowadays (7), the incidence of male infertility has increased, in parallel with a decline in semen quality (8, 9). The etiology and severity of male infertility seem to independently affect reproductive outcomes even under ART settings (7, 10). Moreover, the age of the population seeking ART is increasing steadily as both women and men are postponing childbearing. Aging couples, in turn, poses enormous challenges for clinicians and researchers alike as female age seems to be the central factor for pregnancy success (11).

The success of ART has traditionally been reported as the live birth rate (3). However, widespread use of preimplantation genetic testing (PGT) and embryo cryopreservation in the past two decades has allowed the introduction of alternative metrics of effectiveness. In 2016, the POSEIDON (**P**atient-**O**riented **S**trategies **E**ncompassing **I**ndividualize**D O**ocyte **N**umber) collaborative group proposed a new metric of success in ART, namely, the ability to obtain the number of oocytes needed to achieve at least one euploid blastocyst for transfer (12). Indeed, transfer of euploid embryos markedly reduces the age-related decrease in implantation rates (13–15), thus making the POSEIDON's marker a pragmatic endpoint for clinicians providing care to infertility patients (16).

A clinical predictive model to estimate the number of oocytes needed to achieve at least one euploid embryo for transfer -and that provides a revised estimate of the probability of achieving this outcome when fewer than the predicted number of oocytes are obtained after one or more oocyte retrieval cycles- would be invaluable for both patient counseling and establishment of a working plan with a clear goal for management. We, therefore, assessed the factors influencing embryo ploidy and estimated the predicted probability of blastocyst euploidy as a function of each mature oocyte retrieved. We used mature oocytes in preference over all oocytes as the former are the gametes with the capacity to support embryo development to the blastocyst stage. Then, we developed an integrative predictive model composed of pretreatment risk factors to estimate the minimum number of mature oocytes needed to achieve at least one euploid blastocyst for transfer, with the aim of offering clinicians and patients a counseling tool at the point of care.

## Materials and Methods

This cohort study included consecutive infertile couples attending ANDROFERT Fertility Center in Campinas, Brazil, from February 2016 to June 2017. The Ethics Committee of *Instituto Investiga* approved the study (Approval number 1.913.076; CAAE 64291417.0.0000.5599).

### Study Population and Patients' Eligibility Criteria

We queried our ART database (ClinisysIVF®) for infertile couples who underwent *in vitro* fertilization/intracytoplasmic sperm injection (IVF-ICSI) treatment with the intention to have trophectoderm biopsy for preimplantation genetic testing for aneuploidy (PGT-A). PGT-A was used for reasons of advanced maternal age, severe male factor infertility, recurrent miscarriage, repeated implantation failure, as well as for patients who were concerned about the euploidy status of their embryos. Eligible patients were all consecutive couples undergoing their first treatment cycle in our Clinic irrespective of the protocol used for ovarian stimulation. We only included patients with a complete IVF/ICSI record. Furthermore, the included patients had at least one mature oocyte retrieved. The mature (metaphase II [MII]) oocytes were inseminated for own use and all resulting viable blastocysts were biopsied.

Women who underwent PGT for balanced translocations or single-gene diseases, polar body biopsy, and PGT on day 3 embryos were excluded. Patients who had treatment involving oocyte donation were excluded. We also excluded patients who had PGT-A on frozen-thawed blastocysts and those whose cycles involved insemination using sperm from different sources (e.g., ejaculated and surgically retrieved sperm) or the use of both fresh and frozen-thawed gametes (e.g., fresh and frozen-thawed sperm or fresh and frozen-thawed oocytes).

Baseline characteristics of couples included female and male age, body mass indexes (BMI), infertility duration, infertility factor, presence and type of azoospermia, antral follicle count (AFC), anti-Müllerian hormone (AMH) levels, the presence of poor ovarian reserve (POR), and semen parameters. Treatment characteristics included the type of ovarian stimulation, gonadotropin regimen, total gonadotropin dose, sperm source for ICSI, and gamete status for ICSI. Treatment outcomes included the number of oocytes retrieved, number of mature (MII) oocytes retrieved, number of two-pronuclei (2PN) zygotes, number of blastocysts, and number of euploid blastocysts (Supplementary Table 1).

### Assessment of Infertility Factors and Ovarian Reserve

All included couples were evaluated by both a reproductive endocrinologist and an andrologist as per our institution's protocol. Ovarian reserve was determined by antral follicle count (AFC), which was carried out on the early follicular phase (17), and AMH levels using the modified Beckman Coulter AMH generation II assay (18). A POR was defined according to the Poseidon criteria as AFC < 5 and/or AMH < 1.2 ng/ml (12). Male partners underwent a thorough evaluation, including history, physical examination, semen analysis, hormone profile (serum FSH, LH, and total testosterone), and genetic testing (Yq microdeletions and karyotyping) as appropriate (19). Semen analysis was carried out at our institution's andrology laboratory according to the 2010 World Health Organization manual for the examination of human semen (20, 21). Additionally, assessment of sperm DNA fragmentation (SDF) in fresh ejaculates was carried out in all males, using the sperm chromatin dispersion assay (SCD; Halosperm®; Spain) (22), unless the sperm count was too low for an accurate determination of DNA fragmentation levels. The type of azoospermia was determined by a combination of clinical and laboratory data and confirmed by histological evaluation of testicular biopsy specimens taken during sperm retrieval (23, 24).

### Ovarian Stimulation Protocol

Both the conventional antagonist and minimal stimulation protocols were used for ovarian stimulation (OS). In brief, the antagonist protocol involved subcutaneous (SC) administration of recombinant FSH monotherapy (rec-FSH; Gonal-F®, Merck) or rec-FSH combined with recombinant LH (2:1 ratio rec-FSH and rec-LH; Pergoveris®, Merck). Gonadotropin administration started either on day 2 or day 3 of the cycle after confirmation of absence of ovarian cysts by ultrasound scanning, and a flexible GnRH antagonist regimen was initiated by daily SC administration of 0.25 mg cetrorelix (Cetrotide®, Merck) when the leading follicles achieved 12–14 mm in mean diameter, including the day of trigger (25). The minimal stimulation protocol involved the use of either clomiphene citrate or letrozole early in the cycle followed by a low dose of injectable recombinant gonadotropin. The choice of OS regimen and gonadotropin dosage was based on the clinician's assessment of ovarian reserve, female age, and history of previous response to OS. At our institution, minimal ovarian stimulation is reserved for selected POR patients.

### Trigger and Oocyte Retrieval

Final oocyte maturation was achieved by SC administration of triptorelin 0.2 mg (Decapeptyl®, Ferring) or recombinant hCG (Ovidrel®, Merck). In general, the criterion for trigger included the presence of two follicles of 17 mm or greater. Oocyte retrieval was carried out under transvaginal ultrasound guidance and intravenous sedation with propofol 35–36 h after triggering.

### Laboratory Procedures

The cumulus-corona-oocytes complexes were stripped after exposure to hyaluronidase, classified according to nuclear maturity, and kept in culture at 37°C and 5.5% CO_2_ until sperm microinjection (26). The injected oocytes were incubated for 16–18 h at 37°C under 5.5% CO_2_ and 5% O_2_ until fertilization was confirmed by visualization of 2PN and two polar bodies 16–18 h after insemination. Zygotes were kept in culture to reach the blastocyst stage, and embryo quality was scored according to the criteria described by Gardner (27). Oocyte retrieval, sperm processing, and ICSI were carried out in clean room environments (28).

### Trophectoderm Biopsy and Preimplantation Genetic Testing

PGT-A was performed using trophectoderm cells, which were subjected to next-generation sequencing (NGS) analysis of 24 chromosome copy numbers with the purpose of transferring only euploid embryos. In brief, biopsies were performed on embryos that reached the blastocyst stage on days 5–7 by cutting a small piece of trophectoderm (5–10 cells) with the aid of non-contact diode laser (Octax™, MTG, Germany), as previously described (29). The biopsied fragments were immersed into 0.2 mL PCR tubes in a total volume of 2.5 uL of Tris-EDTA Buffer pH 8.0 (ThermoFisher Scientific Baltics, Vilnius, Lithuania), frozen at −20 Celsius degrees, and shipped to Chromosome laboratory (São Paulo, Brazil) for analysis. Specimens were subjected to cell lysis, whole genome amplification (WGA), and construction of libraries using the Ion Reproseq kit (ThermoFisher Scientific, Germany). The DNA quantity was estimated using StepOne (ThermoFisher Scientific, Germany) following the manufacturer's protocol, and NGS was performed using the Ion Torrent PGM™ platform (ThermoFisher Scientific, Germany). Euploidy data analysis was carried out on the Ion Reporter software version 5.2 calibrated at medium sensitivity, using Low-Coverage Whole-Genome workflow. Copy numbers were measured quantitatively, and embryos were classified according to the PGDIS criteria for reporting embryo results (30). Embryos with <20% of abnormal cells were classified as euploids whereas embryos with >80% of abnormal cells were deemed aneuploid. Mosaic embryos were those with abnormal cells ranging from 20 and 80%.

### Statistical Analysis

Descriptive statistics were calculated for patient demographics and treatment characteristics. We analyzed the distribution of the number of euploid blastocysts per patient to determine how to model our dataset. Then, we determined the influence of a total of 26 pretreatment and treatment predictors on this distribution (Supplementary Table 1). For this, we used the adaptive LASSO (Least Absolute Shrinkage and Selection Operator) method **[**31, 32**]**. Once the predictors were selected, we utilized logistic regression to fit the final model. The binary response was euploidy (yes/no) for each mature oocyte. To assess the effect of predictors on critical intermediate embryonic stages, we conducted separate logistic regression analyses with the binary responses “2PN zygote (yes/no) for each mature oocyte,” “blastocyst (yes/no) for each 2PN zygote,” and “euploid blastocyst (yes/no) for each biopsied blastocyst.”

We made the following assumptions: (i) the embryos are statistically independent concerning the ploidy status, and (ii) the probability of a mature oocyte to reach the blastocyst stage is constant across women, depending only on explanatory variables (predictors) that might affect the response. With these assumptions, the logistic model generates the probability, “p,” as an output, where “p” is the probability that any mature oocyte would turn into a euploid blastocyst, given the relevant predictors. The final model was internally validated using the holdout method. The dataset was randomly partitioned in two, i.e., training and validation data sets. The training dataset size was 80% of the total and it was used for the calculations of the fitting; the validation data set was 20% of the total. The quality of the fit was evaluated by the area under the curve (AUC) of the ROC curve. The effect size of predictors on the blastocyst euploidy probability was calculated as the % decrease in blastocyst euploidy.

The probability of a mature oocyte to become a euploid blastocyst, p, was used to compute the minimum number of mature oocytes (*n*) needed to obtain ≥1 euploid blastocyst, using the formula $n\geq \frac{\log\left(1-\pi \right)}{\log\left(1-p\right)}$. The probability of success was denoted by π. Its complement, 1 − π, is the risk, i.e., the probability of having no euploid blastocyst despite achieving the estimated number of mature oocytes. The 95% confidence intervals for “p” were obtained from the logistic regression. These limits were introduced in the formula for “n,” to generate the corresponding limits of the confidence interval for the required number of mature oocytes. The mathematical operations are valid since the estimators are based on the maximum-likelihood and the functions are monotone. Lastly, we created an online calculator—named “ART Calculator”—to make two types of predictions automatically, using the formula and mathematical equations described above. The first is based on pretreatment predictors to estimate the minimum number of mature oocytes to achieve ≥1 euploid blastocyst for transfer in infertile couples undergoing IVF/ICSI. The second utilizes pretreatment information and the actual number of mature oocytes collected or accumulated to provide a revised estimate of the probability of achieving the aforesaid outcome when fewer than the predicted number of mature oocytes are obtained after one or more oocyte retrieval cycles. Computations were carried out using JMP® PRO 13 (SAS Institute, Cary, North Carolina, US). We adopted an alpha level of <0.05 as significant. The ART Calculator was programmed using Hypertext Preprocessor (PHP) language.

## Results

### Population Characteristics

A total of 347 patients were included, and their demographics and treatment characteristics are reported in Table 1. The mean female age of our selected cohort was 38.9 years (95% confidence interval [CI]: 32.4–42.4 years) with a mean number of mature oocytes retrieved per patient of 6.3 (95% CI: 1.0–12.0). The mean number of blastocysts available for TE biopsy and NGS analysis per patient was 2.1 (95% CI: 0.0–5.0). A total of 2,520 mature oocytes were injected, resulting in 882 blastocysts that were subjected to PGT-A. Overall, the percentage of euploid embryos after NGS in our cohort was 34.8%. The mean number of euploid blastocysts per patient was 0.74 (95% CI: 0.0–2.0). The distribution of the number of euploid blastocysts per woman was found to be the negative binomial (Supplementary Material).

**Table 1:** Characteristics of 347 couples and their treatment at first cycle of intracytoplasmic sperm injection (ICSI) and trophectoderm biopsy for preimplantation genetic testing for aneuploidy (PGT-A).

**Characteristics**	**Mean**	**95% CI**
Infertility duration (years)	7	4–10
Female age (years)	38.9	32.4–42.4
Male age (years)	42.4	35.0–53.0
BMI, female (kg/m^2^)	24.5	20.3–31.2
BMI, male (kg/m^2^)	27.6	23.1–32.3
Infertility factor, *N* (%)		
*Male factor*	117 (33.8)	-
*Unexplained*	63 (18.2)	-
*Endometriosis*	33 (9.5)	-
*Endocrine/Anovulatory*	26 (7.5)	-
*Anatomic/Tubal*	10 (2.9)	-
*>1 type*	98 (28.1)	-
AFC (*n*)	6.7	3-12
AMH (ng/mL)	1.39	0.20-3.00
Semen parameters:		
*Sperm count (M/mL)*	30.5	0.0–79.8
*Total motility (%)*	63.4	43.6–76.0
*Sperm morphology (%)*	2.9	1.0–5.1
*DFI (%)*	21.6	10.0–43.0
Azoospermia, *N* (%)	65 (18.7)	–
*Non-obstructive*	44	–
*Obstructive*	21	
POR, *N* (%)	178 (51.3)	–
Conventional OS; *N* (%):	304 (87.6)	–
*rFSH monotherapy*	111	–
*rFSH+rLH*	193	–
Minimal stimulation, *N* (%)	43 (12.4)	–
Total gonadotropin dose (IU)		
*Conventional*	3,145	1,875–3,300
*Minimal*	525	315–795
Sperm source for ICSI; N (%):		
*Ejaculate*	391 (71.5)	–
*Epididymis*	27 (4.9)	–
*Testicle*	129 (23.6)	–
Gamete status for ICSI; N (%)		
*Fresh, sperm [S] + oocyte [O]*	301 (86.8)	–
*Frozen-thawed, [S + O]*	0 (0.0)	–
*Combined, fresh [S] + frozen-thawed [O]*	7 (2.0)	–
*Combined, frozen-thawed [S] + fresh [O]*	39 (11.2)	–
Oocyte and embryo parameters:		
*No. Oocytes retrieved*	8.2	2.0–16.2
*No. Mature (MII) oocytes*	6.3	1.0–12.0
*%MII oocytes*	78.1	50.0–100.0
*Fertilized oocytes* (2PN)	4.3	1.2–8.2
*%2PN fertilization*	67.3	33.3–100.0
*No. Blastocysts*	2.1	0.0–5.0
*%Blastulation*	48.9	0.0–100.0
*No. Euploid blastocysts*	0.74	0.0–2.0
*%Euploid blastocysts*	34.8	0.0–100.0

*BMI, body mass index; OS, ovarian stimulation; AFC, antral follicle count; AMH, anti-Müllerian hormone; DFI, Sperm DNA fragmentation index; FSH, follicle stimulating hormone; POR, poor ovarian reserve according to POSEIDON criteria; 2PN, two pronuclei zygote; MII, metaphase II*.

### Development of Predictive Model

For the selection of variables, the stopping rule on the LASSO procedure was based on the adjusted Akaike Information Criteria (AIC). The model is a generalized linear model. The response is the number of euploid blastocysts. The negative binomial distribution was chosen for the fit. Accordingly, the link function is the logarithm. For the overdispersion, we chose the identity as the link function. The fitted model selected female age, sperm source used for ICSI—in particular, testicular sperm extracted from men with non-obstructive azoospermia (NOA)—, and the number of mature oocytes as predictors (Supplementary Table 2). Apart from these variables, no significant association was found between the response variable and all other pretreatment and treatment characteristics (Supplementary Table 2).

Female age was to a large extent the most relevant factor for predicting the probability of a blastocyst being euploid given each mature oocyte. The difference in the loglikelihood ascribed to age—adjusted for sperm source—was 30.9 (*df* = 2; *p* < 0.0001). The number of mature oocytes was also significantly associated with the response “≥1 euploid blastocyst,” as expected, due to a positive cohort-size effect. This parameter was included in the final model as part of the response variable in association with blastocyst euploidy.

**Figure 1:**
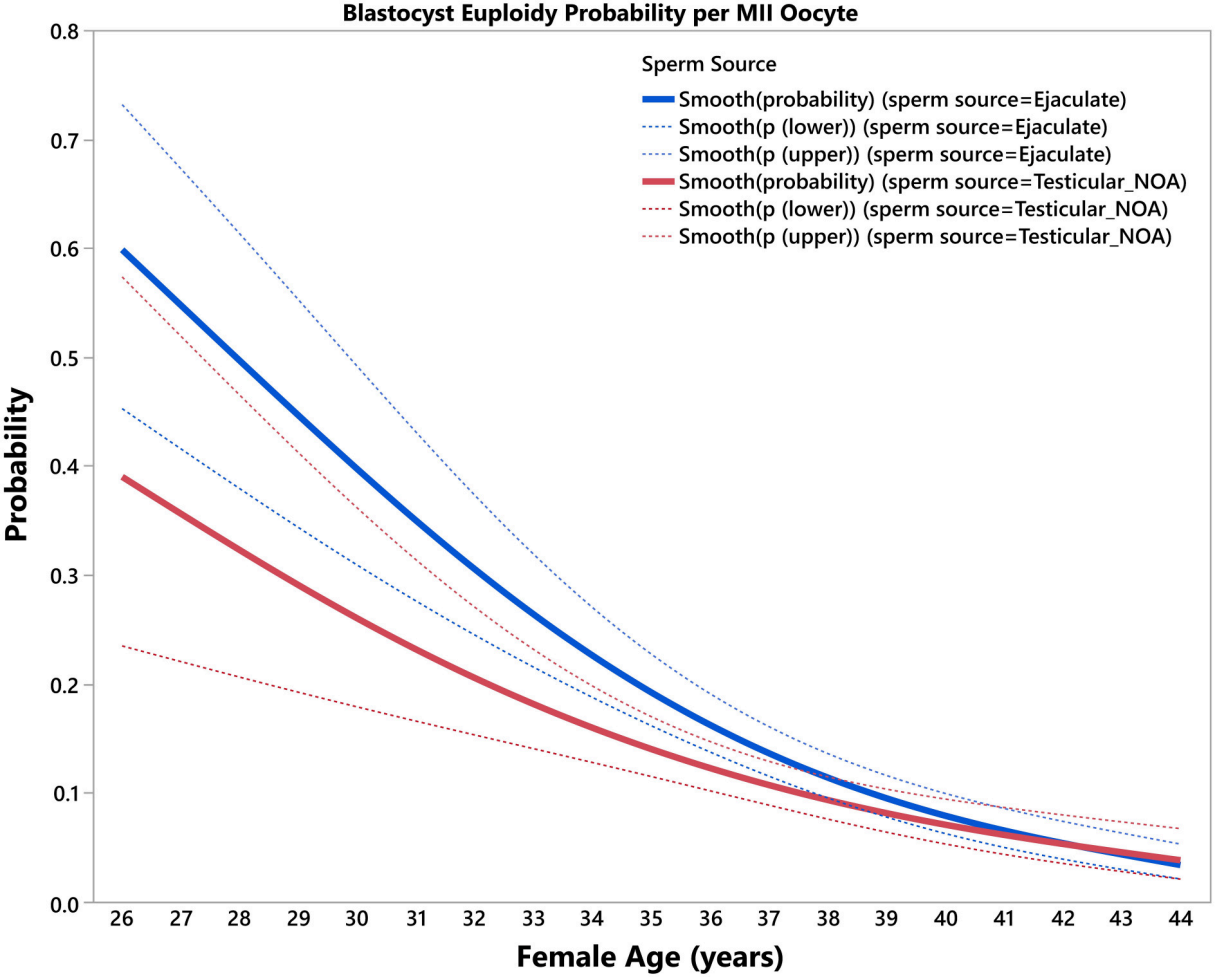
Blastocyst euploidy probability per mature (MII) oocyte. The plots show the probability of a MII oocyte turn into a euploid blastocyst as a function of female age. The estimated probabilities (solid curves) and their 95% confidence interval (dotted curves) are presented according to sperm source to be used for IVF/ICSI, namely, ejaculated sperm (blue) and testicular sperm extracted from patients with non-obstructive azoospermia (NOA) (red). The relations are non-linear and characterized by a differential modulatory effect of sperm source across age (see text).

The final predictive model, based on female age and type of sperm used for ICSI, and its correspondent equation is presented in Table 2. The estimated predicted probabilities of a mature oocyte turning into a euploid blastocyst decreased progressively as a function of female age and were negatively modulated overall by use of testicular sperm from men with NOA across age (Figure 1). The effect size of female age on blastocyst euploidy probability per MII oocyte from year (t) to year (t+1) was defined as the ratio p(t+1)/p(t) × 100. There was a significant decrease (*p* < 0.001) in the probability of a MII oocyte become a euploid blastocyst. The overall yearly reduction in the blastocyst euploidy probability per MII oocyte using ejaculated and testicular sperm were 14.4 and 12.1%, respectively. The loss was progressive with every year of female age but the yearly reduction was not remarkably affected by sperm source (Supplementary Table 3).

**Table 2:** Final model for pred (p) of euploid blastocyst per mature (MII) oocyte.

***Equation Y = a+b [Sperm = “Ejaculate”] +c [Sperm = Ejaculate](FemaleAge-38.9066) + d [Sperm = Testicular_NOA](FemaleAge-38.9066), where*** $p=\left(\frac{1}{1+e^{-y}}\right)$				
**Term**	**Estimate**	**SE**	**Wald ChiSquare**	**Prob >ChiSquare**
(Intercept)	−2.6518	0.1174497	371.96	<0.0001
spermSource [EJACULATE]:(ageFemale-37.9384)	−0.2045457	0.0269435	57.63	<0.0001
spermSource [TESTICULAR_NOA]:(ageFemale-37.9384)	−0.1530924	0.0354465	18.65	<0.0001
spermSource [Ejaculate]	0.2231659	0.1174497	3.61	0.0574
Statistics:
Response: euploid blastocyst given MII oocytes
Distribution: binomial
Estimation method: Nominal logistic fit
Mean model link: Logit
Area under the curve: 0.71589

*The Nominal Logistic Fit was the final model with the best prediction of the probability of ≥1 euploid blastocyst per mature (MII) oocyte. The full equation is written at the top of the table. Each particular characteristic is displayed with an associated P-value (Prob >ChiSquare) giving the indication of how much weight each variable will contribute to the predictive number of mature oocytes. a, intercept; b, spermSource [EJACULATE]; c_**v**_, spermSource [EJACULATE]:(ageFemale-37.9384), d, spermSource [NOA]:(ageFemale-37.9384); SE, standard error*.

Results of logistic regression analyses assessing the effect of predictors on critical intermediate embryonic stages showed that the impact of testicular sperm on the final model depended primarily on its negative effect (*p* < 0.0001) on the probability of obtaining a 2PN zygote per mature oocyte. This effect was independent of female age (Supplementary Material). The overall geometric mean of the reduction in the probability of having a 2PN zygote per MII oocyte by use of testicular sperm over ejaculated sperm was 17%. By contrast, testicular sperm alone had no significant effect on the probability of a 2PN zygote turn into a blastocyst, and the effect was only marginal (*p* = 0.07) on the probability of having a euploid blastocyst per biopsied blastocyst. However, when associated with female age, testicular sperm had a significant negative effect on the probability of having a euploid blastocyst (per biopsied blastocyst) (*p* < 0.0001). In this case, the overall female age-adjusted geometric mean of the reduction in the probability of having a euploid blastocyst (per biopsied blastocyst) by using testicular compared with ejaculated sperm was 24%.

Unlike testicular sperm, female age had no significant effect on the probability of having a 2PN zygote per mature oocyte, but it affected the chances of having both a blastocyst per 2PN zygote (*p* = 0.003) and, more importantly, a euploid blastocyst per biopsied blastocyst (*p* < 0.0001) (Supplementary Material).

### Model Validation and Performance

The model validation was carried out using the holdout sampling method. The AUCs obtained from the fitted model on both datasets—training and validation—were virtually identical, thus confirming that our model was internally validated (Supplementary Material). The predictive ability of the model assessed by the area under the ROC curve was 71.6%.

### Development of Calculator

Using the probabilities generated by our model in conjunction with the formula $n>\frac{\log\left(1-\pi \right)}{\log\left(1-p\right)}$, we created an online calculator to compute the minimum number of mature oocytes needed to obtain ≥1 euploid blastocyst automatically, which can be used at the point of care as a counseling tool and potentially influence decision and management. The calculator computes the value of “p,” given the female age and sperm source. Then, given the value of the accepted risk, that is, 1-π, it uses the formula to compute the minimum number of mature oocytes and its associated uncertainty (95% confidence interval). Pretreatment, the calculator allows the user to set the probability of success and generates the minimum number of mature oocytes needed for at least one euploid blastocyst accordingly. The higher the required probability of success (lower risk), the higher the number of mature oocytes needed to achieve the intended goal. Posttreatment, the calculator estimates the probability of achieving at least one euploid blastocyst when fewer than the predicted number of mature oocytes are obtained after one or more oocyte retrieval cycles. The online calculator is available at https://members.groupposeidon.com/Calculator/.

### Examples of Predicting the Individualized Number of Mature Oocytes Needed for Achieving ≥1 Euploid Blastocyst for Transfer

**Figure 2:**
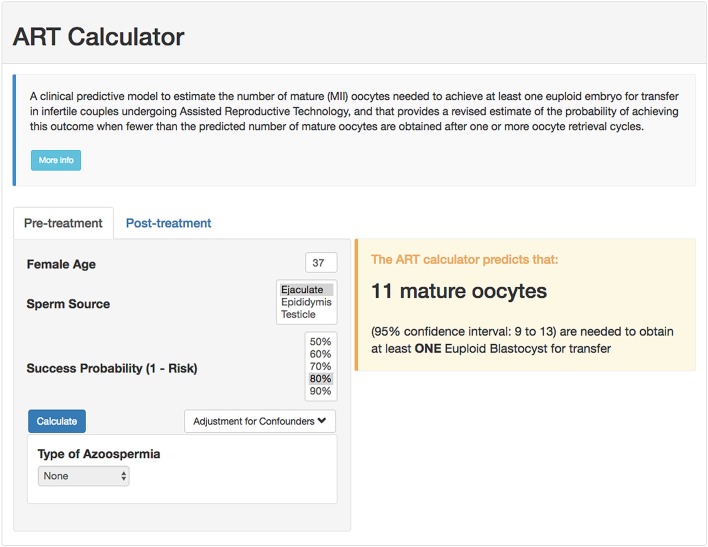
Online calculator to determine the minimum number of mature oocytes required to obtain at least one euploid blastocyst for transfer in infertile patients undergoing IVF/ICSI cycles. The figure shows how the online calculator can be used in an office-based setting. Pretreatment, clinicians should input the patient age and the sperm source to be used for IVF/ICSI. If the option “Testicle” is marked, then the type of azoospermia should be also defined. The probability of success is set by the user and indicates the chance of having ≥1 euploid blastocyst when the predicted number of mature oocytes is achieved. Its complement is the risk, that is, the chance of having no (zero) euploid blastocysts when the predicted number of oocytes is achieved. Once the button “calculate” is pressed, a text box will pop-up on the right side of the screen, indicating the predicted minimum number of mature oocytes needed for obtaining at least one euploid blastocyst, with its 95% confidence interval.

**Figure 3:**
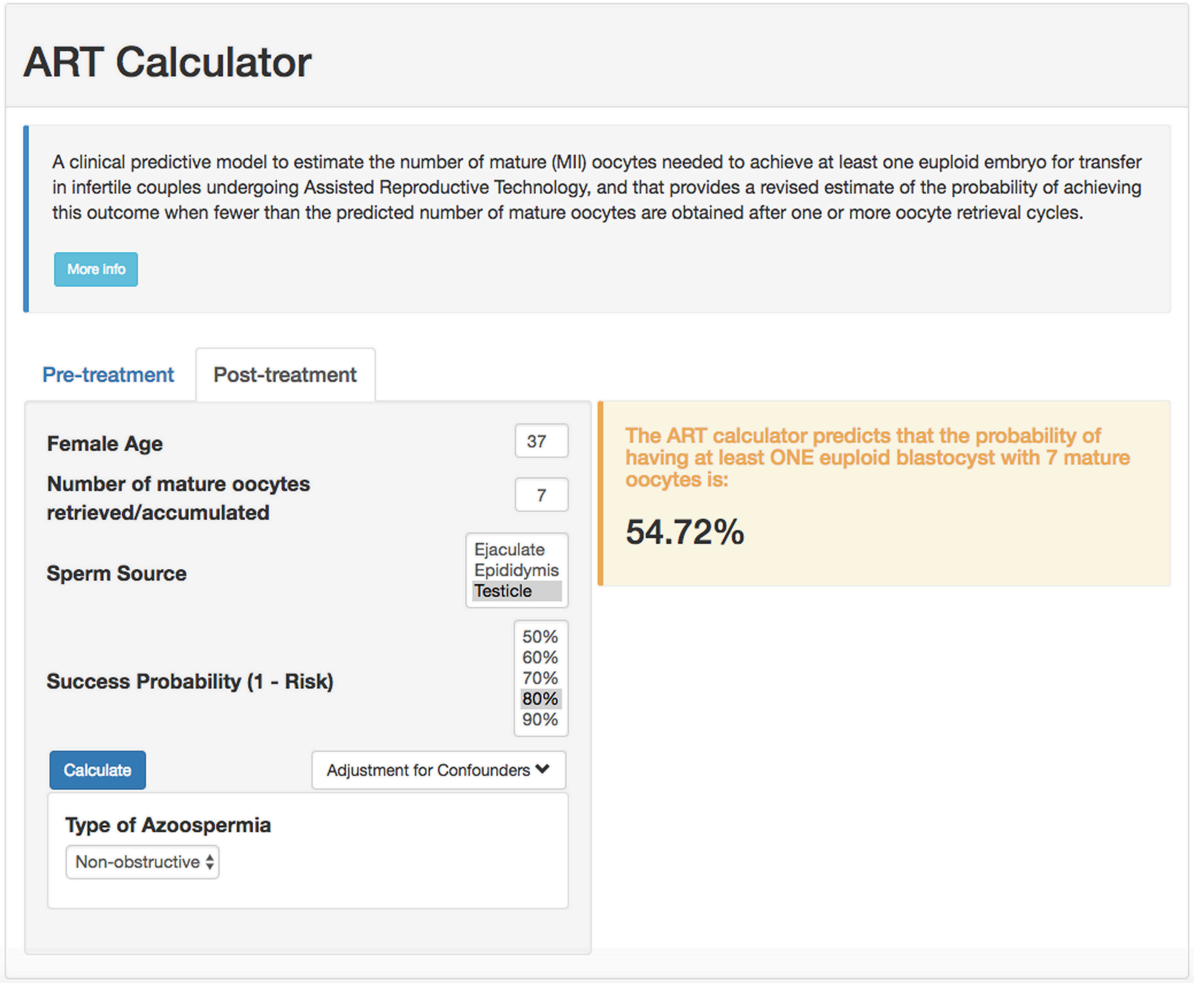
ART online calculator. The figure shows how the online calculator can be used posttreatment, i.e., when fewer than the predicted number of mature oocytes are obtained after one or more oocyte retrieval cycles. Clinicians should input the pretreatment information and the actual number of mature oocytes collected or accumulated. The probability of success is set by the user; it reflects the chance that the estimation is correct given the number of oocytes input. Once the button “calculate” is pressed, a text box will pop-up on the right side of the screen, indicating the predicted probability of achieving ≥1 euploid blastocyst with the number of mature oocytes available.

As an example, for a probability of 80% of success set by the user, i.e., 20% risk of having zero euploid blastocyst, a patient of 37 years-old undergoing IVF/ICSI who will use ejaculated sperm from her partner needs a minimum of 11 (confidence interval: 9–13) mature oocytes to obtain at least one euploid blastocyst for transfer (screenshot, Figure 2). The computation means that this predicted number of mature oocytes has a chance of 80% of success (or 20% risk of failure) in achieving at least one euploid blastocyst. By contrast, if the same patient utilizes testicular sperm for ICSI from a partner with NOA, the minimum number of mature oocytes will be 14 (confidence interval: 11–17), assuming the same probability of success. If this hypothetical patient had seven mature oocytes collected, then the revised estimates concerning the probability of having at least one euploid blastocyst would be ~64 and 55% for ejaculated and testicular sperm, respectively (screenshot; Figure 3).

**Figure 4:**
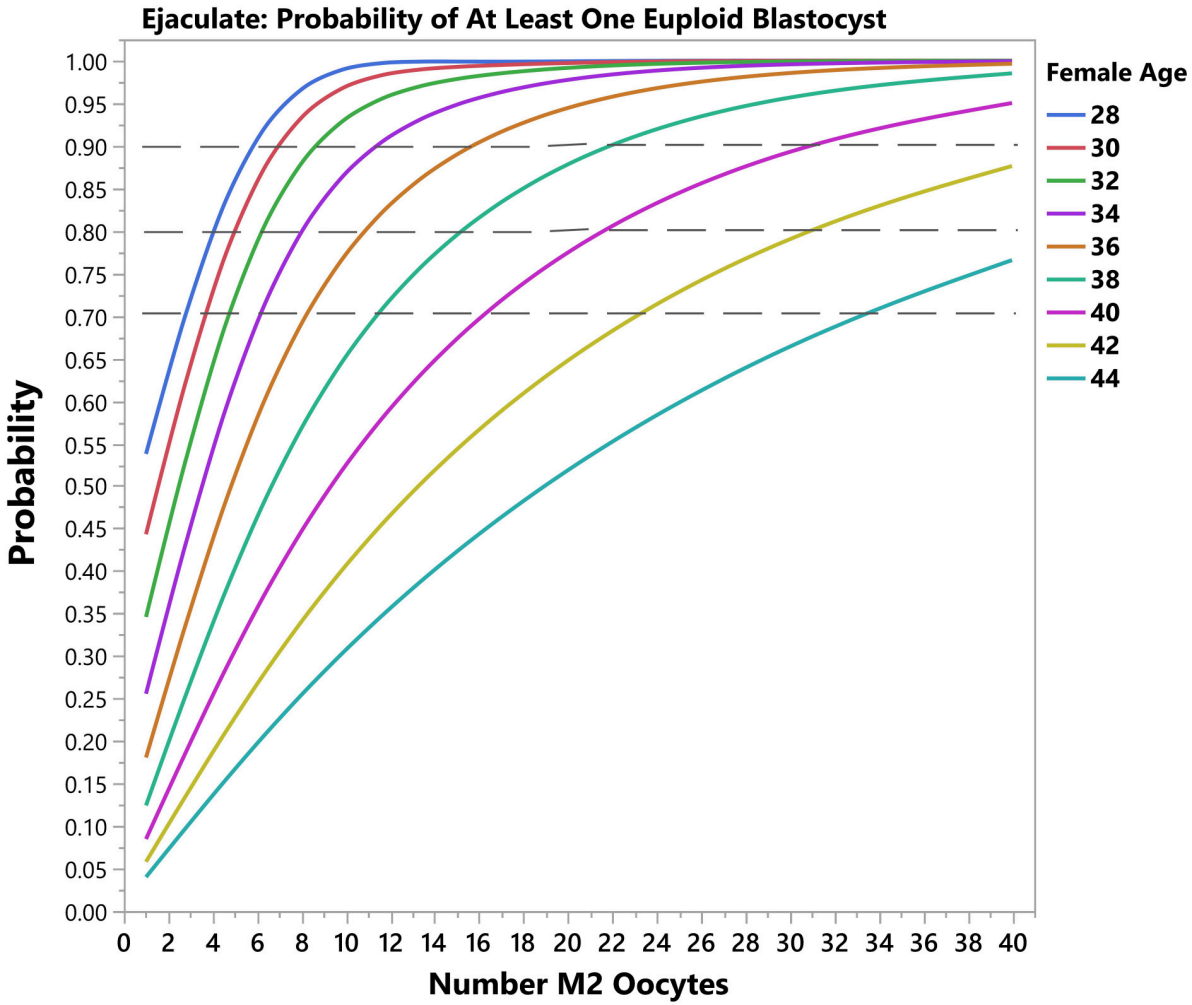
Predictive model output (Ejaculated sperm). The plots show the predicted probability of having ≥1 euploid blastocyst oocyte according to the number of mature oocytes. Each solid curve represents a female age category. The dotted reference lines indicate the 70, 80, and 90% bands for achieving the desired outcome.

**Figure 5:**
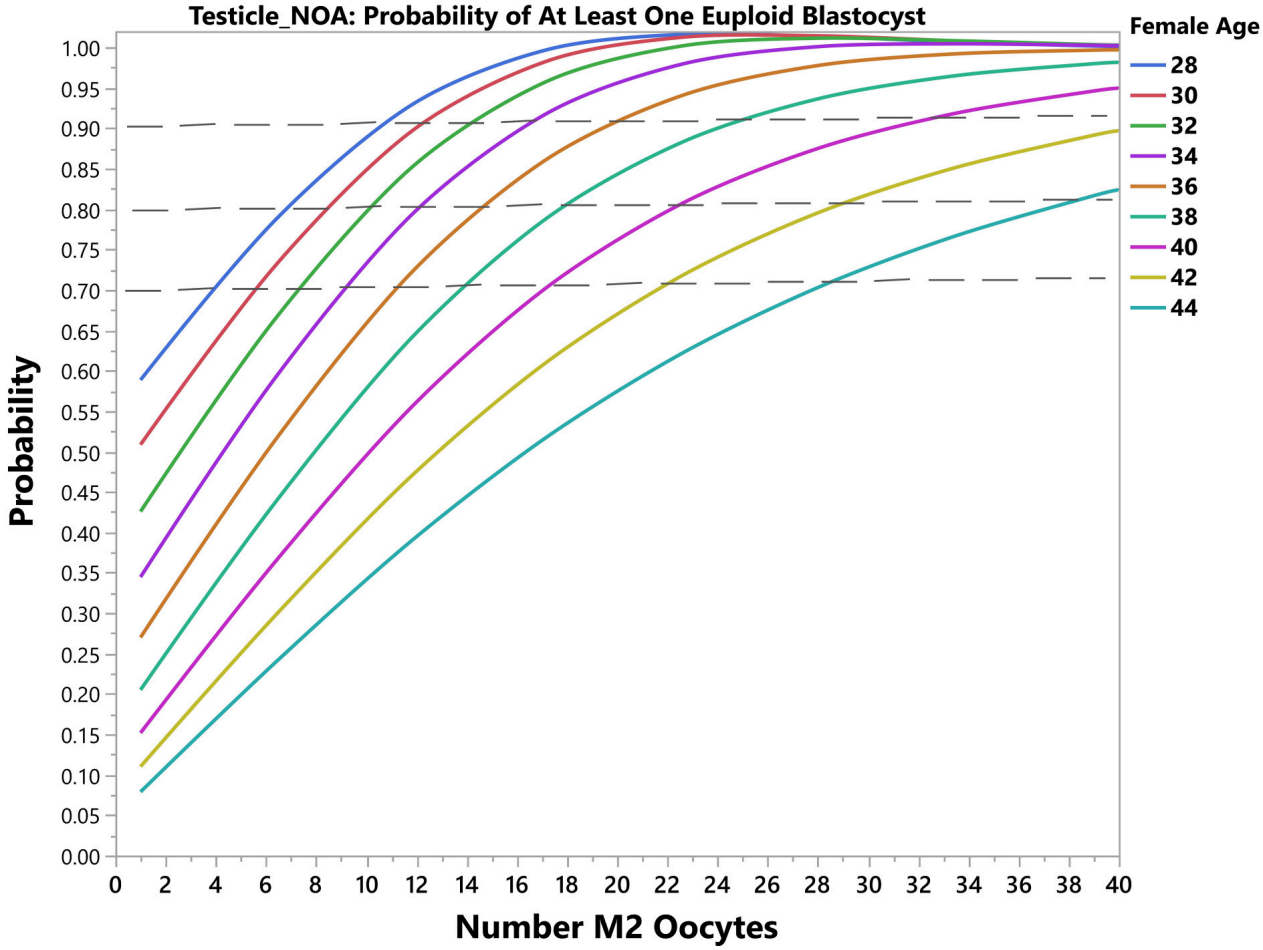
Predictive model output (Testicular sperm from non-obstructive azoospermic [NOA] men). The plots show the predicted probability of having ≥1 euploid blastocyst oocyte according to the number of mature oocytes. Each solid curve represents a female age category. The dotted reference lines indicate the 70, 80, and 90% bands for achieving the desired outcome.

Using another example, for a probability of 90% of success set by the user, i.e., 10% risk of zero euploid blastocyst, a patient of 30 years-old will need a minimum of 4 (band interval: 3–6) mature oocytes to obtain at least one blastocyst for transfer by use of ejaculated sperm for ICSI. The predicted number of mature oocytes will be 7 (confidence interval: 5–11) if testicular sperm is used. In this case, the prediction indicates a chance of 90% of success in achieving at least one euploid blastocyst. Like the previous case, the revised probability of having at least one euploid blastocyst can be obtained. If she then had 3 mature oocytes collected, the revised estimates concerning the probability of having at least one euploid blastocyst would be 78 and 55% for ejaculated and testicular sperm, respectively. Figures 4, 5 depict the probability curves to obtain at least one euploid blastocyst according to the number of mature oocytes for different age groups and sperm sources.

## Discussion

The primary goal of our prediction model was to allow the development of a calculator to provide individualized pretreatment estimates concerning the number of mature oocytes needed to achieve ≥1 euploid blastocyst for transfer in infertile couples undergoing IVF/ICSI treatment. We found that the age of the woman was by far the most critical predictor for the likelihood of achieving ≥1 euploid blastocyst for transfer. Aside from woman's age, sperm source for ICSI, in particular testicular sperm obtained from men with non-obstructive azoospermia, resulted in a response lower than with the use of ejaculated sperm for all female ages. Based on these variables, we developed a predictive model to estimate the individualized probability of blastocyst euploidy per each mature oocyte retrieved. Our results indicate that the estimated probability of a mature oocyte turn into a euploid blastocyst decreases progressively with aging, and sperm source exerts a modulatory effect. Specifically, the use of testicular sperm from men with NOA negatively modulates the probability of a mature oocyte become a euploid blastocyst overall. After model internal validation, we developed a mathematical equation to compute the individualized minimum number of mature oocytes needed to achieve at least one euploid blastocyst for transfer based on the predicted probabilities. Lastly, we created a calculator to make these computations automatically.

Our foremost motivation for conducting this study was to develop a clinical tool to objectively estimate the POSEIDON's marker of success in ART, namely, “the ability to retrieve the number of oocytes needed to obtain at least one euploid embryo for transfer in each patient” (12, 31). The number of oocytes needed to achieve at least one euploid embryo is a logical endpoint that could help clinicians to both counsel their patients more effectively and plan treatment with the mindset to achieve the individualized oocyte number (16, 32). Although live birth rate (LBR) is the preferable endpoint in couples undergoing ART, it depends on a multitude of controlled and uncontrolled factors, thus making it challenging for individualized predictions about the number of oocytes needed to achieve the desired outcome.

Our model relied essentially on analysis of an ICSI dataset from infertile couples who have undergone PGT-A using NGS analysis. This design seems ideal as the outputs of the whole IVF process were obtained for analysis. We used ≥1 euploid blastocyst as a dependent variable due to the importance of such variable for ART success. Indeed, ~50–60% of euploid blastocysts implant across all age categories, thus indicating that availability of a euploid blastocyst for transfer may offset to a great extent the adverse effect of increased female age on pregnancy success (14). Currently, analysis of embryo genetic status is carried out by a variety of methods using blastocyst trophectoderm cells, which largely replaced fluorescence *in situ* hybridization (FISH) analysis of cleavage-stage blastomere cells, as they provide reliable information on the copy numbers of all 24 chromosomes. Among the existing methods, recent reports suggest that next-generation sequencing (NGS) has the highest accuracy (13–15). Euploidy rates by NGS between trophectoderm cells (TE) and embryo inner cell mass (ICM) are similar, with a low (~3%) rate of clinically relevant non-concordance between a mosaic TE and a euploid ICM (33).

Importantly, our prediction tool does not imply by any means that PGT-A should be carried out routinely. Naturally, we included only cycles with PGT-A because the model development was based on the dependent variable “euploid blastocyst ≥1.” Therefore, information about blastocyst genetic status had to be available for calculating the probability that a mature oocyte would become a euploid blastocyst. Clinicians willing to use the ART Calculator do not have to provide PGT-A data nor do they need to offer PGT-A to their patients unless they wish to confirm the results of the ART Calculator in their settings.

We also used mature oocytes as a response variable since these are the gametes with the capacity to support embryo development to the blastocyst stage and live birth. In ART, ovarian stimulation using exogenous gonadotropins is routinely applied to promote the growth of multiple follicles. Human chorionic gonadotropin (hCG) or GnRHa are the commonly used agents for triggering final oocyte maturation, which can be administered alone or combined in different dose schemes (34). Following trigger, immature “metaphase I” oocytes progress to the mature “metaphase II” stage of development (35). During this process, the first polar body is extruded, thus allowing diploid cells to turn into haploid gametes that attain competence for fertilization by spermatozoa. After the trigger, oocyte retrieval should be precisely timed to enable the effective retrieval of mature oocytes. However, several issues might affect the proportion of mature oocytes available for fertilization. As examples, short duration of OS, reduced follicle size on day of trigger, short time interval between trigger and oocyte retrieval, and patient errors in timing or injection technique, as well as problems in absorption, can contribute, alone or combined, to reduced mature oocyte output (36, 37). In our study, we avoided these confounding factors by modeling predictors as a function of mature oocytes to increase the generalizability of our prediction model.

### Interpretation

Not surprisingly, we found that female age was the most important variable to predict the likelihood of embryo euploidy, thus corroborating previous reports (29, 38, 39). In a recent study, we estimated the age-related decrease in the probability of blastocyst euploidy—calculated per biopsied blastocyst—using NGS data from fresh trophectoderm human cells (29). We observed that the geometric mean of the yearly decrease variation in the probability of a blastocyst being euploid was 13.6%, but the effect was progressive with every year of female age, varying from 1.2% in women below the age of 30 to over 15% in those older than 39 years. In the study mentioned above, we also found that blastocyst cohort size had an impact on the likelihood of having at least one euploid embryo for transfer across all age groups. The present study confirms these findings using mature oocytes. Indeed, with aging, there is an increase in both oocyte chromosomal abnormalities and cytoplasmic dysfunctions, as well as a progressive reduction in the number of primordial follicles (40). As a result, both embryo quantity and quality are reduced, thus explaining the reasons why IVF success is lower in older women than in younger counterparts (41).

The source of sperm used for ICSI also affected the chances of achieving ≥1 euploid blastocyst for transfer in infertile couples undergoing ART. In particular, we found that use of testicular sperm extracted from men with NOA had a negative modulatory effect. However, the effect of sperm source on the blastocyst euploidy probability per mature oocyte was markedly dependent on female age. Despite significant in younger patients, the impact of testicular sperm from men with NOA was virtually offset in women of 40 years and over. According to our results, the chances of mature oocytes turning into euploid blastocysts are below 8% in such patients and are negligible after the age of 44. These observations indicate that in these patients the negative influence of age on embryo quality is so dramatic that it cannot be changed further by any factor, including the sperm source. By contrast, ejaculated sperm, epididymal or testicular sperm from men with obstructive azoospermia (OA), and testicular sperm from non-azoospermic men with high sperm DNA fragmentation had no apparent adverse effect on the number of euploid blastocysts. Our results are consistent with previous reports which showed that pregnancy success by ICSI is differentially affected by both sperm source and type of azoospermia (23, 26). In general, men with NOA who have their sperm used for ICSI are at a reproductive disadvantage (23). The reasons are not entirely known but might be related to the fact that testicular specimens from NOA men have higher rates of DNA fragmentation and aneuploidy than both ejaculated and epididymal/testicular counterparts from other male infertility categories (42, 43). Hence, critical embryonic stages might be affected by using such sperm for ICSI, including zygote and embryo development, thus decreasing both the number and genetic quality of resulting blastocysts (44).

In the present study, we showed that the likelihood of obtaining ≥1 euploid blastocyst depended on the number of retrieved mature oocytes. These findings confirm previous observations showing that the proportion of IVF/ICSI patients with at least one euploid blastocyst for transfer depends on female age and blastocyst cohort size (29, 38, 39). Moreover, they are consistent with the overall positive association between oocyte number and delivery rates (45, 46), especially when the cumulative live birth rates are computed (47). Our results indicate that for any given probability of blastocyst euploidy, the higher the number of MII oocytes the higher the chances of having at least one euploid blastocyst within the patient embryo cohort, an effect that was modulated by female age and sperm source used for ICSI. Measuring the effect size of predictors, we found that the blastocyst euploidy probability was reduced by approximately 14% and 12% for every year of female age overall when ejaculated and testicular sperm were used, respectively, but the magnitude of loss was differentially affected by age (Supplementary Table 3). A 30-year-old patient will lose about 10% in this probability in a year, whereas the loss is about 1.5x higher in a patient aged 40. In mathematical terms, although a euploid blastocyst may be achieved in women older than 40 at the expense of high oocyte numbers (Figures 3, 4), this may be unrealistic in clinical practice as well as prohibitively costly. Indeed, it has been suggested that the added benefit of increasing the number of oocytes in women older than 41 using current therapeutic strategies is limited, and should be discouraged in women older than 43 years (48).

### Clinical Importance

To our knowledge, this is the first pretreatment model to estimate the individualized number of oocytes needed to obtain at least one euploid blastocyst for transfer in infertile couples undergoing IVF/ICSI. By converting our model into a calculator, healthcare providers can estimate such numbers automatically. Our model is primarily intended to be a counseling tool for shaping expectations of couples before embarking on ART. However, it may also be used to help clinicians design individualized patient-oriented treatment strategies aiming at obtaining the number of mature oocytes needed for achieving ≥1 euploid blastocyst for transfer. For example, assuming a risk of 20%, the minimum number of mature oocytes for at least one euploid blastocyst in a couple whose woman is aged 37 and the male partner has viable sperm in his ejaculate varies from 9 to 13. This goal is feasible to achieve using individualized conventional OS in women with normal or high ovarian reserve, unlike in poor ovarian reserve patients (32, 49, 50). In the latter, the clinician might consider alternative OS protocols involving oocyte or embryo accumulation (51, 52). By contrast, given the conditions as above, the predicted number of mature oocytes varies from 2 to 4 in a young patient of 30 years-old. In such a case, even in the presence of low ovarian reserve, the clinician might achieve the intended goal using a single OS cycle and thus advise the patient accordingly. Along the same lines, in patients with adequate pre-stimulation ovarian parameters who had a suboptimal ovarian response in a previous cycle of conventional OS (e.g., Poseidon's groups 1 and 2), estimating the individualized oocyte number might help clinicians to explore pharmacological interventions aimed at increasing the oocyte yield (49, 50, 53). In older patients with low ovarian reserve, the predicted oocyte number might be tough to achieve even after using the best OS protocol and multiple oocyte retrievals, especially when the partners have NOA and testicular sperm are to be used for ICSI. In such cases, our model allows patients and clinicians to make informed decisions based on the predicted number of oocytes needed to obtain at least one euploid blastocyst for transfer. Along the same lines, posttreatment, i.e., after the retrieval of less than the predicted number of mature oocytes, the ART calculator provides invaluable information about the likelihood of achieving a euploid blastocyst, thus allowing transparent discussion and shared-decision making.

According to our model, in women of the same age, the probability of a mature oocyte turn into a euploid blastocyst is reduced if testicular sperm from a partner with NOA were used for ICSI. The aforesaid negative effect of testicular sperm was also noted when intermediate responses were analyzed separately, in particular, the “2PN zygote probability per mature oocyte,” and to a lesser extent the “blastocyst euploid probability per biopsied blastocyst.” This means that the observed effect of testicular sperm on blastocyst euploidy is due to a combined adverse effect across critical embryonic steps, mainly the fertilization stage. As a result, the final number of blastocysts available for transfer is reduced, thus affecting the likelihood of having at least one euploid blastocyst within the patient embryo cohort. Thus, in such cases, the number of mature oocytes has to be adjusted to account for the loss during the IVF process. Notably, our data indicate that the negative effect of testicular sperm was only remarkable in ICSI cycles involving men with NOA, corroborating other reports (44). By contrast, the use of testicular sperm from men with obstructive azoospermia or non-azoospermic patients with high DFI was not associated with the probability of blastocyst euploidy per mature oocyte. Indeed, previous reports indicate that in these cases testicular sperm perform optimally for ICSI (54–56). The possible reasons for lack of any detrimental effect by use of testicular sperm from men with high DFI and OA are that these cells have lower sperm DNA fragmentation rates than ejaculated counterparts (55–57). Moreover, unlike NOA, spermatogenesis in men with OA is not disrupted (24, 54).

On the other hand, female age had no significant effect on the probability of a MII oocyte turn into a zygote. However, the age of the woman markedly affected the subsequent embryonic stages, in particular, the probability of a blastocyst turning into a euploid blastocyst, thus indicating that the age-related decrease in the probability of each mature oocyte turning into a euploid blastocyst is intrinsically related to both oocyte and embryo quality (40). In women aged 40 years and over, in whom the impact of age on oocyte and embryo quality is so remarkable, the negative effect of testicular sperm on the blastocyst euploidy probability per mature oocyte is virtually lost (Figure 1).

### Strengths and Limitations

Many studies produced models to predict live birth after a single or multiple IVF/ICSI cycles (11, 58–60). However, no model like ours exists to predict the minimum number of mature oocytes needed to achieve at least one euploid blastocyst for transfer. Although models predicting live birth are useful for counseling purposes, they do not provide a target goal for clinical management. In contrast, our predictive model is intended to serve both as a useful clinical tool for counseling infertile couples and to guiding clinicians to most optimally treat the patient with the mindset to achieve the individualized oocyte number. Another study has suggested that in addition to female age, ovarian biomarkers, in particular, AMH, could influence the chances of obtaining euploid embryos (39). In this study, the authors used a univariate regression analysis to identify variables with a tendency of association with the primary outcome. Then, these variables were included in the multivariate analysis, which showed that female age and AMH were independently associated with the rate of euploid blastocysts. However, information about how regression analyses were modeled concerning the distribution of the number of euploid blastocysts and the impact of the source of sperm and type of azoospermia were not available.

A critical question when developing predictive models is to determine the variables that best describe the response variable. We have chosen the LASSO statistical method because the procedure allows for simultaneous estimation and variable selection by applying a shrinking (regularization) process that penalizes the coefficients of the regression variables (61). As a result, it removes not only redundant variables but also discovers relevant predictive variables, thus minimizing prediction error. Internal validation showed that the predictive ability of our model was accurate, thus confirming previous observations that the LASSO method is a powerful tool for selecting a reduced number of explanatory variables to describe a response variable (62, 63). The method is, therefore, advantageous as it not only makes the model easier to interpret but also enables algorithms to work faster and reduce overfitting. Furthermore, we assessed the distribution of the number of euploid blastocysts, and here we report for the first time that this distribution follows a negative binomial. Applying the correct distribution is critical to most optimally select the model for statistical analysis; if a wrong assumption concerning the response variable is taken, the generalizability of the prediction model is undermined (64).

Since our model was developed using retrospective data from a single ART Clinic, there is a need to validate its prediction ability externally to confirm generalizability. Along these lines, our estimations cannot be generalized to IVF patients undergoing cleavage-stage embryo transfer as our study is based on blastocyst biopsies and NGS analysis. Also, we did not assess the accuracy of our estimations using other genetic analysis platforms. Lastly, the effect of cycle number and other OS regimens were not analyzed. Our model should be used with caution to decide whether a patient should undergo fertility treatment.

### Future Research

External multi-center validation is currently ongoing using suitable ART datasets from different countries. If required, model calibration using external datasets will be carried out as a means to increase performance and generalizability.

## Conclusion

We developed an internally validated pretreatment model to predict the minimum number of mature oocytes needed to obtain at least one euploid blastocyst for transfer in infertile couples undergoing IVF/ICSI. The model was used to create a novel calculator to make the predictions automatically. This tool will help healthcare providers to counsel infertility patients concerning the individualized oocyte number needed to optimize the chances of having a euploid blastocyst for transfer, thus shaping patients' expectations. Also, the model may have utility to guide clinicians on a risk-shared decision analysis about ART treatment options aimed at achieving the individualized oocyte number, although further external validation is required.

## Author Contributions

SE designed and coordinated the study. JC carried out the statistical analyses and helped with data interpretation. FB coordinated data collection and extraction, helped with the study design and data interpretation. JS coordinated the development of the ART Calculator prototype and its hosting platform. All authors contributed to drafting and critical discussions, revised, and accepted the final manuscript.

### Conflict of Interest Statement

The authors declare that the research was conducted in the absence of any commercial or financial relationships that could be construed as a potential conflict of interest. The handling editor is currently co-organizing a Research Topic with one of the authors SE, and confirms the absence of any other collaboration.
