# Supplementary material for: A Novel Predictive Model to Estimate the Number of Mature Oocytes Required for Obtaining at Least One Euploid Blastocyst for Transfer in Couples Undergoing in vitro Fertilization/Intracytoplasmic Sperm Injection: The ART Calculator
Source: Front Endocrinol (Lausanne). 2019 Feb 28;10:99. doi: 10.3389/fendo.2019.00099 (PMC6403136; doi:10.3389/fendo.2019.00099)
Supplement: Supplementary file 1 [file Table_1.DOCX]

**Supplementary Table 1**. Patient and treatment variables in dataset

|  | **Type of variable (description)** |
| --- | --- |
| **A. Patient characteristics** | |
| Female age | Numerical/continuous (years) |
| Male age | Numerical/continuous (years) |
| Female body mass index | Numerical/continuous (kg/m^2^) |
| Male body mass index | Numerical/continuous (kg/m^2^) |
| Infertility duration | Numerical/continuous (years) |
| Infertility factor | Categorical/nominal (male factor, female factor, >1 type, unexplained) |
| Female infertility etiology | Categorical/nominal (endometriosis, endocrine/anovulatory, anatomic/tubal, unexplained, other, >1 type) |
| Baseline FSH level | Numerical/continuous (n) |
| Antral follicle count | Numerical/continuous (n) |
| Anti-Müllerian hormone | Numerical/continuous (ng/ml) |
| Poor ovarian reserve | Categorical/nominal (yes, no, undefined) |
| Associated male factor | Categorical/nominal (yes, no, undefined) |
| Sperm count | Numerical/continuous (x10^6^/ml) |
| Sperm motility (total) | Numerical/continuous (%) |
| Sperm morphology | Numerical/continuous (%) |
| Sperm DNA fragmentation | Numerical/continuous (%) |
| Presence of azoospermia | Categorical/nominal (yes, no, undefined) |
| Type of azoospermia | Categorical/nominal (obstructive, non-obstructive) |
| **B. Treatment characteristics** | |
| Type of ovarian stimulation | Categorical/nominal (conventional, minimal) |
| Type of gonadotropin used in conventional OS | Categorical/nominal (rFSH, rFSH+rLH) |
| Gonadotropin dose (total) | Numerical/continuous (IU) |
| Sperm source for ICSI | Categorical/nominal (ejaculate, epididymis, testicle) |
| Sperm status for ICSI | Categorical/nominal (fresh, frozen-thawed) |
| Ejaculated sperm for ICSI | Categorical/nominal (homologous/abnormal, homologous/normal, heterologous) |
| Oocyte status | Categorical/nominal (fresh, frozen-thawed) |
| **C. Outcome variables** | |
| No. oocytes retrieved | Numerical/continuous (n) |
| No. MII oocytes retrieved | Numerical/continuous (n) |
| No. 2PN zygotes | Numerical/continuous (n) |
| No. blastocysts | Numerical/continuous (n) |
| PGT-A result | Categorical/nominal (euploid, aneuploid, mosaic) |
| No. euploid blastocysts | Numerical/continuous (n) |

FSH: follicle-stimulating hormone; ICSI: intracytoplasmic sperm injection; IU: international units; OS: ovarian stimulation; PGT-A: preimplantation genetic screening for aneuploidy; rFSH: recombinant FSH; rLH: recombinant LH; 2PN: two pronuclei; MII: metaphase II;
