# Supplementary material for: A Novel Predictive Model to Estimate the Number of Mature Oocytes Required for Obtaining at Least One Euploid Blastocyst for Transfer in Couples Undergoing in vitro Fertilization/Intracytoplasmic Sperm Injection: The ART Calculator
Source: Front Endocrinol (Lausanne). 2019 Feb 28;10:99. doi: 10.3389/fendo.2019.00099 (PMC6403136; doi:10.3389/fendo.2019.00099)
Supplement: Supplementary file 2 [file Table_2.DOCX]

**Supplementary Table 2.** Adaptive Lasso regression analysis for determining the effect of co-variates on the number of euploid blastocysts

| *Term* | *Estimate* | *SE* | *Wald ChiSquare* | *Prob > ChiSquare* | *Lower 95%* | *Upper 95%* |
| --- | --- | --- | --- | --- | --- | --- |
| (Intercept) | -1.586739 | 0.8191673 | 3.7520627 | 0.0527 | -3.192278 | 0.0187992 |
| SpermSource [Ejaculate-Testicular/NOA]:femaleAge-39.4904) | -0.203824 | 0.048617 | 17.576549 | **<0.0001** | -0.299111 | -0.108536 |
| SpermSource [Testicular/NOA-Testicular/NOA]:femaleAge-39.4904) | 0 | 0 | 0 | 1.0000 | 0 | 0 |
| Male age | 0 | 0 | 0 | 1.0000 | 0 | 0 |
| BMI, Female | 0 | 0 | 0 | 1.0000 | 0 | 0 |
| BMI, Male | 0.0157388 | 0.0280832 | 0.314088 | 0.5752 | -0.039303 | 0.0707809 |
| AMH | 0 | 0 | 0 | 1.0000 | 0 | 0 |
| AFC | 0 | 0 | 0 | 1.0000 | 0 | 0 |
| Baseline FSH | 0 | 0 | 0 | 1.0000 | 0 | 0 |
| Female infertility [Combined-Unexplained] | 0 | 0 | 0 | 1.0000 | 0 | 0 |
| Female infertility [Female-Unexplained] | 0 | 0 | 0 | 1.0000 | 0 | 0 |
| Female infertility [Male-Unexplained] | 0 | 0 | 0 | 1.0000 | 0 | 0 |
| Female infertility [Anatomic-None] | 0 | 0 | 0 | 1.0000 | 0 | 0 |
| Female infertility [Anatomic-Endocrine-None] | 0 | 0 | 0 | 1.0000 | 0 | 0 |
| Female infertility [Anatomic-Endometriosis-None] | 0 | 0 | 0 | 1.0000 | 0 | 0 |
| Female infertility [Endocrine-None] | 0 | 0 | 0 | 1.0000 | 0 | 0 |
| Female infertility [Endometriosis-None] | 0.2729445 | 0.4579799 | 0.3551859 | 0.5512 | -0.62468 | 1.1705686 |
| Female infertility [Endocrine-Endometriosis-None] | -0.129911 | 0.7986794 | 0.0264573 | 0.8708 | -1.695294 | 1.4354721 |
| POR associated | 0 | 0 | 0 | 1.0000 | 0 | 0 |
| Male factor associated | 0 | 0 | 0 | 1.0000 | 0 | 0 |
| Semen profile | 0 | 0 | 0 | 1.0000 | 0 | 0 |
| Sperm count | 0 | 0 | 0 | 1.0000 | 0 | 0 |
| Sperm motility | 0 | 0 | 0 | 1.0000 | 0 | 0 |
| Sperm morphology | 0 | 0 | 0 | 1.0000 | 0 | 0 |
| Sperm DNA fragmentation index (DFI) | 0 | 0 | 0 | 1.0000 | 0 | 0 |
| OS type [CONVENTIONAL-MINIMAL] | 0 | 0 | 0 | 1.0000 | 0 | 0 |
| Gonadotropins [recFSH+recLH-None] | 0 | 0 | 0 | 1.0000 | 0 | 0 |
| Gonadotropins [recFSH alone-recFSH+recLH] | 0 | 0 | 0 | 1.0000 | 0 | 0 |
| Gonadotropins [recFSH alone-None] | 0 | 0 | 0 | 1.0000 | 0 | 0 |
| Gonadotropin dose | 0 | 0 | 0 | 1.0000 | 0 | 0 |
| Sperm status for ICSI [FRESH-FROZEN-THAWED] | 0 | 0 | 0 | 1.0000 | 0 | 0 |
| Oocyte status [FRESH-FROZEN-THAWED] | 0 | 0 | 0 | 1.0000 | 0 | 0 |
| MII oocytes | 0.0809248 | 0.0162602 | 24.769037 | **<0.0001** | 0.0490553 | 0.1127943 |
| SpermSource [Ejaculate-Testicular/NOA] | 0 | 0 | 0 | 1.0000 | 0 | 0 |
| SpermSource [Ejaculate-Testicular/Other] | 0 | 0 | 0 | 1.0000 | 0 | 0 |
| Dispersion | 0.00001109 | 0.173831 | 4.067e-7 | 0.9995 | -0.340592 | 0.3408134 |
|  |  |  |  |  |  |  |
| *Statistics:*  Response: ≥1 euploid blastocysts  Distribution: negative binomial  Estimation method: Adaptive Lasso with validation column  Mean model link: Log  Dispersion model link: Identity | | | | | | |

SE = standard error
