# Supplementary material for: A Novel Predictive Model to Estimate the Number of Mature Oocytes Required for Obtaining at Least One Euploid Blastocyst for Transfer in Couples Undergoing in vitro Fertilization/Intracytoplasmic Sperm Injection: The ART Calculator
Source: Front Endocrinol (Lausanne). 2019 Feb 28;10:99. doi: 10.3389/fendo.2019.00099 (PMC6403136; doi:10.3389/fendo.2019.00099)
Supplement: Supplementary file 3 [file Table_3.DOCX]

**Supplementary Table 3**. Effect size of female age and sperm source on blastocyst euploidy probability per mature oocyte. The effect size from year (t) to year (t+1) was defined as the ratio p(t+1)/p(t)x100.

|  | | Loss in the probability that a mature oocyte become a euploid blastocyst per each year of female age |
| --- | --- | --- |
| Sperm Source | Female Age (years) | Geometric Mean (%) |
| Ejaculate | 26 | . |
|  | 27 | 8.3% |
|  | 28 | 9.3% |
|  | 29 | 10.2% |
|  | 30 | 11.1% |
|  | 31 | 12.0% |
|  | 32 | 12.9% |
|  | 33 | 13.6% |
|  | 34 | 14.3% |
|  | 35 | 14.9% |
|  | 36 | 15.5% |
|  | 37 | 15.9% |
|  | 38 | 16.4% |
|  | 39 | 16.7% |
|  | 40 | 17.0% |
|  | 41 | 17.3% |
|  | 42 | 17.5% |
|  | 43 | 17.7% |
|  | 44 | 17.8% |
| Testicular (NOA) | 26 | . |
|  | 27 | 9.1% |
|  | 28 | 9.6% |
|  | 29 | 10.1% |
|  | 30 | 10.5% |
|  | 31 | 10.9% |
|  | 32 | 11.3% |
|  | 33 | 11.6% |
|  | 34 | 11.9% |
|  | 35 | 12.2% |
|  | 36 | 12.4% |
|  | 37 | 12.7% |
|  | 38 | 12.9% |
|  | 39 | 13.0% |
|  | 40 | 13.2% |
|  | 41 | 13.3% |
|  | 42 | 13.4% |
|  | 43 | 13.5% |
|  | 44 | 13.6% |
