# Supplementary material for: A Novel Predictive Model to Estimate the Number of Mature Oocytes Required for Obtaining at Least One Euploid Blastocyst for Transfer in Couples Undergoing in vitro Fertilization/Intracytoplasmic Sperm Injection: The ART Calculator
Source: Front Endocrinol (Lausanne). 2019 Feb 28;10:99. doi: 10.3389/fendo.2019.00099 (PMC6403136; doi:10.3389/fendo.2019.00099)
Supplement: Supplementary file 4 [file Data_Sheet_1.docx]

**Supplemental Material**

**1. Distribution of the number of euploid blastocysts in dataset**

The distribution of the number of euploid blastocysts in dataset followed a negative binomial. This is evidenced by the univariate statistical analysis, where a gamma-Poisson distribution fits very well to the sample distribution (Supplementary material; Tables 1 and 2).

Table 1 (Supplementary material). Univariate statistical analysis of the number of euploid blastocyst distribution

| Type | Parameter | Estimate | Lower 95% | Upper 95% |
| --- | --- | --- | --- | --- |
| Location | Lambda | 0.7354086 | 0.5922671 | 0.9153501 |
| Overdispersion | Sigma | 2.2924108 | 1.7883531 | 3.0878009 |

-2log(Likelihood) = 602.220688826818

Table 2 (Supplementary material). Descriptive distribution

| 100.0% | maximum | 7 |
| --- | --- | --- |
| 99.5% |  | 6.71 |
| 97.5% |  | 4.55 |
| 90.0% |  | 2 |
| 75.0% | quartile | 1 |
| 50.0% | median | 0 |
| 25.0% | quartile | 0 |
| 10.0% |  | 0 |
| 2.5% |  | 0 |
| 0.5% |  | 0 |
| 0.0% | minimum | 0 |

The histogram is depicted in Figure 1 below. In red, the fitted density function. The two parameters, location and dispersion of the distribution are shown in the graph (Figure 1).


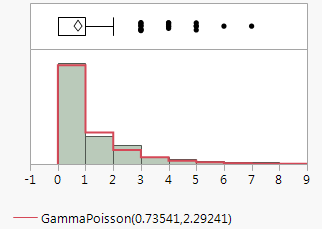


Figure 1: Histogram and fitted density function

Figure 2 (below) shows the probability plot whose linearity evidences the appropriateness of the negative binomial.


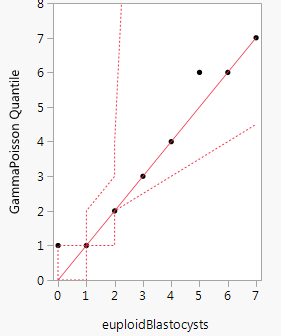

Figure 2: Probability plot

Reference

Samuel Kotz, N. Balakrishnan and Normal L. Johnson: Univariate Discrete Distributions, 3rd Edition. 2005 John Wiley & Sons, Inc.

**2. Logistic regression analyses assessing the impact of predictors on critical intermediate embryonic stages**

2.1. 2PN Fertilization

| Term |  | Estimate | Std Error | ChiSquare | Prob>ChiSq |
| --- | --- | --- | --- | --- | --- |
| Intercept |  | 0.66637318 | 0.0632325 | 111.06 | **<.0001*** |
| spermSource3[Ejaculate]:(ageFemale-37.9384) |  | -0.0199504 | 0.0195641 | 1.04 | 0.3078 |
| spermSource3[NOA]:(ageFemale-37.9384) |  | 0.00969795 | 0.0227356 | 0.18 | 0.6697 |
| spermSource3[Ejaculate] |  | 0.24906748 | 0.0632325 | 15.52 | **<.0001** |

*Statistics:*

Response: 2PN zygotes given MII oocytes

Distribution: binomial

Estimation method: Nominal logistic fit

Mean model link: Logit

2.2. Blastocyst development

| Term |  | Estimate | Std Error | ChiSquare | Prob>ChiSq |
| --- | --- | --- | --- | --- | --- |
| Intercept |  | -0.0452627 | 0.0764097 | 0.35 | 0.5536 |
| spermSource3[Ejaculate]:(ageFemale-37.9365) |  | -0.0634871 | 0.0211948 | 8.97 | **0.0027*** |
| spermSource3[NOA]:(ageFemale-37.9365) |  | -0.0284706 | 0.0293043 | 0.94 | 0.3313 |
| spermSource3[Ejaculate] |  | -0.0527213 | 0.0764097 | 0.48 | 0.4902 |

*Statistics:*

Response: Blastocyst given 2PN zygotes

Distribution: binomial

Estimation method: Nominal logistic fit

Mean model link: Logit

2.3. Euploid blastocyst

| Term |  | Estimate | Std Error | ChiSquare | Prob>ChiSq |
| --- | --- | --- | --- | --- | --- |
| Intercept |  | -0.7530825 | 0.1352808 | 30.99 | **<.0001*** |
| spermSource3[Ejaculate]:(ageFemale-37.5458) |  | -0.2667254 | 0.0393648 | 45.91 | **<.0001*** |
| spermSource3[NOA]:(ageFemale-37.5458) |  | -0.274332 | 0.0591872 | 21.48 | **<.0001*** |
| spermSource3[Ejaculate] |  | 0.24368926 | 0.1352808 | 3.24 | 0.0716 |

*Statistics:*

Response: Blastocyst given 2PN zygotes

Distribution: binomial

Estimation method: Nominal logistic fit

Mean model link: Logit

**3. Internal Validation**

The final model (see Table 1; text) was validated by the holdout method (80% of the data in the training dataset, 20% on the validation data set). The AUCs for the training and validation datasets were 0.7136 and 0.7036, respectively. The similarity between the ROC curves indicates the model is internally validated (see 3.1 and 3.2). Computations were carried out using JMP Pro 13.

3.1. Receiver Operating Characteristics on Training Data

|  | **eu** | **Area** |
| --- | --- | --- |
|  | 1 | 0.7136 |
|  | 0 | 0.7136 |

3.2. Receiver Operating Characteristics on Validation Data

|  | **eu** | **Area** |
| --- | --- | --- |
|  | 1 | 0.7036 |
|  | 0 | 0.7036 |

3.3. Statistics

| Measure | Training | Validation |
| --- | --- | --- |
| Number of rows | 2016 | 504 |
| Mean -Log p | 0.3502 | 0.3076 |
| Generalized RSquare | 0.1022 | 0.1205 |
| Entropy RSquare | 0.0737 | 0.0903 |
| RMSE | 0.3233 | 0.2959 |
| Mean Abs Dev | 0.2073 | 0.1847 |
| Misclassification rate | 0.1257 | 0.1060 |
